# Anesthetic action on extra-synaptic receptors: effects in neural population models of EEG activity

**DOI:** 10.3389/fnsys.2014.00232

**Published:** 2014-12-10

**Authors:** Meysam Hashemi, Axel Hutt, Jamie Sleigh

**Affiliations:** ^1^INRIA CR Nancy - Grand Est, Team NeurosysVillers-les-Nancy, France; ^2^Department of Anaesthesiology, Waikato Clinical School, University of AucklandHamilton, New Zealand

**Keywords:** GABA receptor, neural mass, propofol, power spectrum, general anesthesia

## Abstract

The role of extra-synaptic receptors in the regulation of excitation and inhibition in the brain has attracted increasing attention. Because activity in the extra-synaptic receptors plays a role in regulating the level of excitation and inhibition in the brain, they may be important in determining the level of consciousness. This paper reviews briefly the literature on extra-synaptic GABA and NMDA receptors and their affinity to anesthetic drugs. We propose a neural population model that illustrates how the effect of the anesthetic drug propofol on GABAergic extra-synaptic receptors results in changes in neural population activity and the electroencephalogram (EEG). Our results show that increased tonic inhibition in inhibitory cortical neurons cause a dramatic increase in the power of both δ− and α− bands. Conversely, the effects of increased tonic inhibition in cortical excitatory neurons and thalamic relay neurons have the opposite effect and decrease the power in these bands. The increased δ-activity is in accord with observed data for deepening propofol anesthesia; but is absolutely dependent on the inclusion of extrasynaptic (tonic) GABA action in the model.

## 1. Introduction

General anesthesia is used daily to enable surgery, but its underlying mechanisms of action are still largely a mystery. In recent decades there have been successful efforts to reveal the drug action on single receptors (Franks and Lieb, 1994; Alkire et al., 2008; Brickley and Mody, 2012), however their effect on neural populations, networks of neural populations, and brain areas, still remains unsolved. To explain the underlying neural mechanism during the loss of consciousness, two prominent hypotheses are the loss of integration information, developed by Tononi (Tononi, 2004; Murphy et al., 2011; Boly et al., 2012), and a sharp phase transition of the brain activity involving a drop of neural activity, put forward by Steyn-Ross et al. (Steyn-Ross et al., 2004; Friedman et al., 2010). These hypotheses are not mutually exclusive. For instance, a recent experimental study on the effects of propofol on neural activity measured at various spatial scales (Lewis et al., 2012) has revealed both decreased functional connectivity between brain areas and a dramatic drop of neuron firing rates after loss of consciousness. A large amount of experimental literature has revealed characteristic spectral signal changes in electroencephalographic data (EEG) and Local Field Potentials (LFPs) during general anesthesia (Cimenser et al., 2011; Lewis et al., 2012; Purdon et al., 2012; Sellers et al., 2013; Vizuete et al., 2014). Moreover, several previous theoretical studies have proposed neural models to explain certain EEG signal features observed during anesthesia (Steyn-Ross et al., 1999, 2013; Bojak and Liley, 2005; Wilson et al., 2006; Foster et al., 2008; McCarthy et al., 2008; Ching et al., 2010, 2012; Hutt, 2013; Liley and Walsh, 2013; Hutt et al., 2013; Hutt and Buhry, 2014). Although these studies may incorporate realistic neurobiological details of the brains' network topology and neuronal function, they have simplified dramatically the anesthetic action by considering only synaptic excitatory and inhibitory receptors. There is a growing amount of experimental research that has revealed the importance of extra-synaptic receptors (ESR) for neural interactions in general (Brickley and Mody, 2012; Hardingham and Bading, 2012), and for anesthesia especially, see Alkire et al. (2008); Hutt (2012) and references therein.

To elucidate the role of ESR in the context of anesthesia, one approach might be to do a theoretical study of a realistic neural population model which reproduces the characteristic signal features observed in EEG. To perform such a theoretical study, it is necessary to incorporate physiological properties of extrasynaptic receptors into neural population models.

Gamma-aminobutyric acid (GABA) receptors are a large and important class of inotropic receptors. These receptors are located in the neuron's membrane and respond to the neurotransmitter GABA by opening Cl^−^ channels and inducing an inward hyperpolarizing membrane current. This response may either be: phasic at synaptic receptors or, tonic at ESR which lie distant from synaptic terminals (Kaneda et al., 1995; Brickley et al., 1996; Semyanov et al., 2003, 2004; Yeung et al., 2003; Belelli et al., 2009). The phasic response evolves on a time scale of 10–200 ms whereas tonic response evolves on a much longer time scale (Hamann et al., 2002; Cavalier et al., 2005).

The precise biochemical origin of tonic inhibition is still heavily debated (Farrant and Nusser, 2005; Bright et al., 2011). A rather simple and intuitive model explains the tonic current as a spillover of excess neurotransmitter from synapses. This is due to incomplete GABA uptake by nearby synaptic GABA_*A*_-receptors. The remaining neurotransmitter is thus able to diffuse to more distant GABA_*A*_-receptors via extracellular space (Nusser et al., 1997; Semyanov et al., 2004; Farrant and Nusser, 2005; Bright et al., 2007). This spillover may explain the longer time scale of tonic responses found experimentally. In addition, this explanation implies that even small concentrations of neurotransmitters are sufficient to generate tonic activity because of the high sensitivity of ESRs.

The effect of ESRs on the dendritic activity has not attracted much attention. This may because there are only a relatively small number of such receptors as compared to synaptic receptors (Kopanitsa, 1997; Farrant and Nusser, 2005). Moreover, only recently have experimental studies been able to classify and localize different sub-types of GABA_*A*_ receptors (Semyanov et al., 2004; Farrant and Nusser, 2005). GABA_*A*_ receptors are pentameric ligand-gated ion channels and it has been found that δ−sub units of GABA_*A*_ receptors occur exclusively at ESRs (Nusser et al., 1998; Wei et al., 2003; Farrant and Nusser, 2005; Belelli et al., 2009; Ye et al., 2013). This indicates a specific role of these receptors for the neural information processing in general with specific implications in diseases (Brickley and Mody, 2012) and consciousness (Kopanitsa, 1997).

Tonic inhibition induced by extra-synaptic GABA_*A*_-receptors represents a persistent increase in the cell membrane's conductance. On the single neuron level, this diminishes the membrane time constant and, consequently, reduces the size and duration of excitatory post-synaptic potentials propagating on the dendrite. Hence tonic inhibition reduces the excitability of the membrane and increases the effective firing threshold (Farrant and Nusser, 2005). At the neural population level, ESRs affect the excitability of interneuron-pyramidal cell networks and thus modify network oscillations (Semyanov et al., 2003). Kopanitsa (1997) argues that the sustained spatially widespread tonic inhibition is energetically more effective for the system to diminish neural population activity than short-lasting local phasic inhibition, since lower neurotransmitter concentrations are sufficient. The critical factor in this mechanism is the the relatively high sensitivity of ESRs to modulations by anesthetic agents (Yeung et al., 2003; Farrant and Nusser, 2005; Orser, 2006; Houston et al., 2012). The brain areas that have been shown to be affected by anesthetic-induced tonic inhibition are the hippocampus (Bai et al., 2001), brain stem (McDougall et al., 2008), cerebellum (Houston et al., 2012), and the thalamus (Belelli et al., 2009). Since these areas are supposed to play a role in general anesthesia (Alkire et al., 2008), ESRs may mediate clinical anesthetic effects, such as hypnosis and amnesia (Kretschmannova et al., 2013). Thus, it is reasonable to argue that GABA_*A*_ ESRs set the background inhibition of neural populations and the brain network and mediate slow consciousness phenomena, such as loss of consciousness, sleep or arousal (Kopanitsa, 1997).

Converse to GABAergic receptors, N-methyl-D-aspartate (NMDA) receptors respond to the neurotransmitter glutamate by excitatory inward Na^+^ and Ca^2+^ currents and K^+^ outward currents. The response of NMDA receptors to glutamate depends on their spatial location with respect to synaptic terminals and the presence of co-agonists. A recent experimental study has revealed that the population of NMDA receptors, which are close to synaptic terminals, are primarily activated by the co-agonist d-serine in the presence of glutamate; while extra-synaptic NMDA receptors (more distant from the synaptic terminals) respond primarily to glutamate and the co-agonist glycine (Mothet et al., 2000; Papouin et al., 2012). D-serine and glycine are endogenous amino acids found naturally in the brain (d-serine is a derivative of glycine). Similar to GABAergic ESRs, it has been shown that there exists a significant ambient glutamate concentration which induces a tonic excitatory current (Sah et al., 1989; Fleming et al., 2011). This current is evoked primarily at extra-synaptic NMDA receptors (Le Meur et al., 2007) and may be regulated by other cells, such as neighboring astrocytes (Panatier et al., 2006; Fleming et al., 2011) which control glutamate uptake and also synthesize d-serine (Wolosker et al., 1999).

Commonly-used GABAergic anesthetic drugs directly modify the corresponding receptors. However, various anesthetics are also known to affect the endogenous co-agonists of NMDA receptors (Martin et al., 1995; Daniels and Roberts, 1998; Papouin et al., 2012). Hence, the possible anesthetic effect on NMDA receptors is more complex and indirect than for GABAergic ESRs. There is a large class of NMDA receptor antagonists, that inhibit the excitatory action of NMDA receptors. These anesthetics induce so-called dissociative anesthesia (Pender, 1970) leading to amnesia and analgesia without depressing respiration, but also characterized by distorted perceptions of sight and sound and feelings of dissociation from the environment. An example of a dissociative anesthetic drug is the inhalational anesthetic xenon which—amongst other actions—binds primarily to the extra-synaptic glycine site of NMDA receptors (Dickinson et al., 2007) and attenuates long-term potentiation present in the hippocampus by reducing extrasynaptic receptor currents (Kratzer et al., 2012).

To understand how the anesthetic effect of ESR activity on the microscopic single neuron scale could lead to changes in EEG and behavior that can be observed at macroscopic scales, it is necessary to establish a bridge between the two scales. This bridge may be formulated as a dynamical theoretical model. Neural population models represent a good candidate for a dynamic description of neural activity at an intermediate mesoscopic scale (Coombes, 2006; Nunez and Srinivasan, 2006; Bressloff, 2012). These models describe properties of ensembles of neurons, such as the mean firing rate and the mean dendritic current (Hutt, 2009), whilst their output variables can be strongly linked to macroscopic experimental quantities such as Local Field Potentials (LFPs) and EEG (Wright and Kydd, 1992; Nunez and Srinivasan, 2006). An increasing number of theoretical studies have used neural population models to describe signal features in LFPs and EEG observed during anesthesia (Foster et al., 2008; Hutt et al., 2013). Most of these studies take into account anesthetic action on excitatory and/or inhibitory synapses (Steyn-Ross et al., 2004; Liley and Bojak, 2005; Hutt and Longtin, 2009; Ching et al., 2010; Hindriks and van Putten, 2012) while few consider ESRs (Talavera et al., 2009). This link between the synaptic receptor properties in an ensemble of neurons and the average population dynamics is straight-forward, since classical neural population models already involve the average synaptic response function. The situation is different for ESRs, since their action is not incorporated into the classical models. A very recent work has filled this gap (Hutt and Buhry, 2014). This theoretical work demonstrated a method to include mathematically extra-synaptic GABA_*A*_ receptor action in neural population models; which enables researchers to study how changing anesthetic ESR action modifies spectral features in the EEG, which might then be observed experimentally.

The current work uses a thalamo-cortical neural population model involving anesthetic synaptic inhibition with a well-established connection topology; and then extends this model by including the effects of extra-synaptic GABAergic receptor action in the presence of the anesthetic drug propofol. With the help of this model, we demonstrate the role of extra-synaptic GABAergic inhibition, and the importance of tonic inhibition in the cortical inhibitory neuronal population, in explaining experimental EEG power spectra.

## 2. Materials and methods

### 2.1. EEG data

We re-analyzed previously-obtained experimental data from subjects that had been given a short propofol anesthetic. The details of the methods can be found in Johnson et al. (2003). In brief, after obtaining regional ethical committee approval and written informed consent, five healthy subjects (mean age 27.7 yrs, four males) were studied. They were on no psychoactive drugs and had been starved for at least 6 h prior to the study. They were monitored and managed as per clinical anesthesia, according to the Australia and New Zealand College of Anesthetists best practice guidelines. The induction consisted of an intravenous infusion of propofol at 1500 mg/hr until the subject no longer responded to verbal command. Typically this occurred about 5 min into the infusion. The estimated effect-site concentrations of propofol were calculated using standard population-based pharmacokinetics models.

The EEG was acquired using the Electrical Geodesics 128 channel Ag/AgCl electrode system (Eugene, CO, USA) referenced to Cz. Electrode impedances were below 30 KOhm (100 MOhm input impedance amplifier). The sampling frequency was 250 Hz, with a 0.1–100 Hz analog band pass filter, and A–D conversion was at 12 bits precision. The EEG data were re-referenced to a grand mean, and band-pass filtered using 3-rd order Butterworth filters 0.2–45 Hz to eliminate line-noise. An additional Whittaker filter was applied to reduce movement and blink artifacts. The power in each frequency was obtained applying a short-time Fourier transform with a moving window of 60 s and 54 s overlap. The power spectra have been computed 1 min before infusion start (*t* = 1 min) and 4 min after infusion (*t* = 5 min). For visualization reasons, these power spectra at different time instances have been smoothed by a running average over frequencies with a 1 Hz window and a 0.017 Hz frequency step.

### 2.2. Thalamo-cortical model

**Figure 1:**
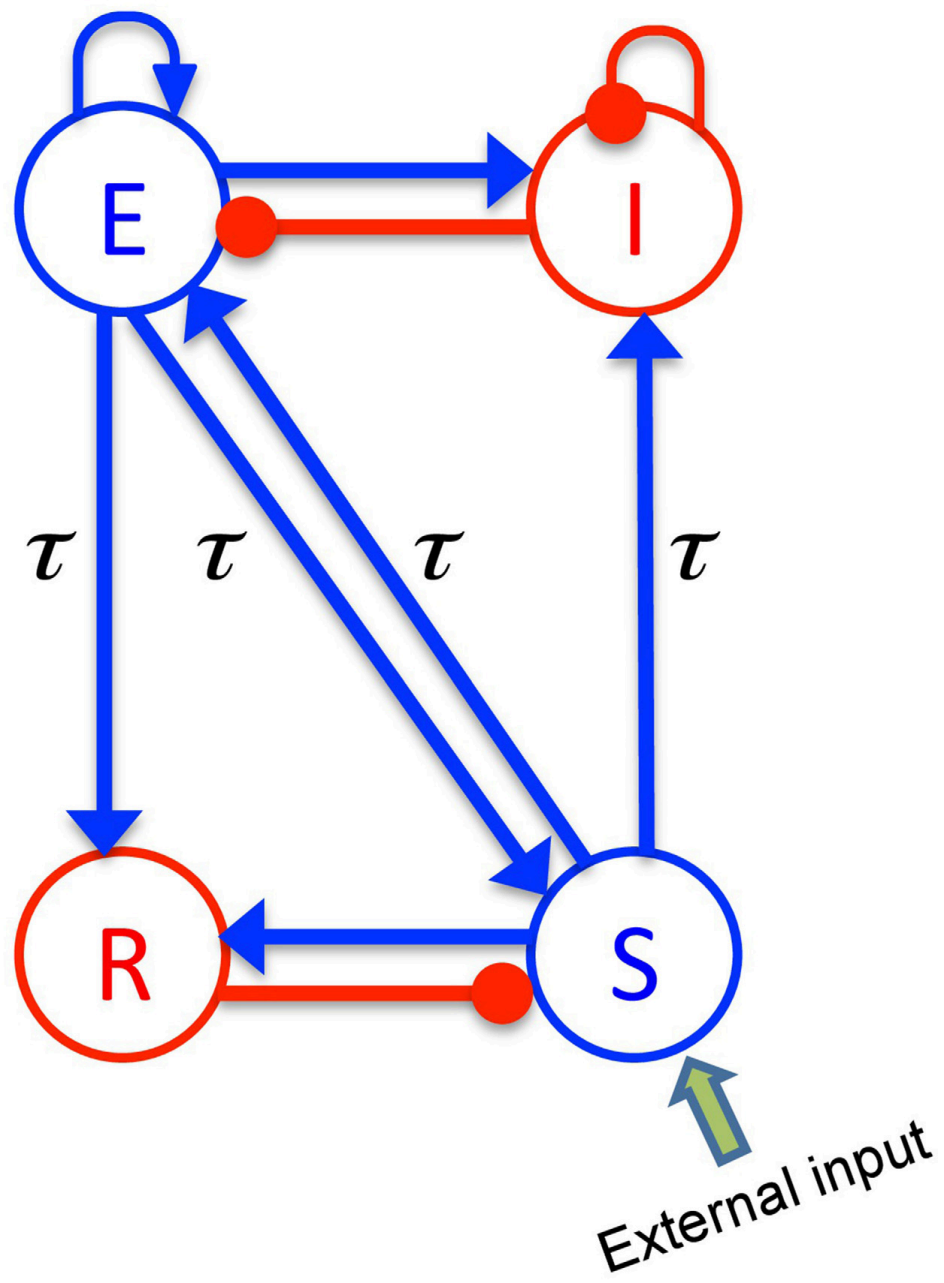
**The schematic of a thalamo-cortical module**. The blue arrows indicate excitatory connections while the red connections with filled circle ends denote inhibitory connections. The symbols *E*, *I*, *S*, and *R* denote the excitatory and inhibitory cortical neurons, thalamo-cortical relay, and thalamic reticular neurons, respectively. In addition thalamocortical and corticothalamic connections exhibit the same nonzero time delay τ.

The body of the model (Robinson et al., 2001; Rennie et al., 2002) is based on a population-level description of a single thalamo-cortical module comprising four populations of neurons, namely excitatory (E) and inhibitory (I) cortical population, a population built of thalamo-cortical relay neurons (S) and of thalamic reticular neurons (R), as shown in Figure 1. The details of the model and the nominal parameter values are taken from a previous work (Robinson et al., 2001, 2002). This model is based on the original idea of Lopes da Silva et al., stating that the α-rhythm represents the noisy thalamic input signal band-pass filtered by feedback-connected cortical and thalamic neural populations (Lopes da Silva et al., 1974). Here we just briefly describe the key concepts of the model. The average soma membrane potential denoted by *V_a_*, for *a* = *E, I, S, R* is modeled by

(1) $$V_{a}(t)=\sum_{b\;=\;E,I,R,S}h_{b}(t)⊗\nu_{ab}\phi_{b}(t-\tau_{ab}),$$

where ⊗ represents the temporal convolution, *h_b_*(*t*) = *H_b_$\bar{h}$_b_*(*t*) where *$\bar{h}$_b_*(*t*) denotes the mean synaptic response function defined by

(2) $${\bar{h}}_{b}(t)=\frac{\alpha \beta_{b}}{\alpha -\beta_{b}}\left(e^{-\beta_{b}t}-e^{-\alpha t}\right),$$

and α and β_*b*_ (with units Hz) are the synaptic rise and decay rates of the synaptic response, respectively. The synaptic decay rates and synaptic response functions depend on the source neurons of type *b* only and are independent of the target neurons. The constant pre-factor *H_b_* defines the response function amplitude subject to the anesthetic concentration. Here, we assume identical excitatory synaptic receptors with constant rise and decay rate. Inhibitory synaptic receptors are also assumed to exhibit identical constant rise and decay rates while their decay rates depend on the anesthetic concentration. This strong approximation is taken from a previous study (Hindriks and van Putten, 2012) to be able to compare our results, while preliminary studies with more realistic parameters show similar results (not shown).

The constants ν_*ab*_ are the strengths of the connections from population of type *b* to population of type *a* (in mVs), and ϕ_*b*_ is the average firing rate of the population *b* (in Hz). The connections between cortex and thalamus are associated with a same nonzero time delay, τ_*ab*_ = τ, while the delay term is assumed to be zero within the cortex and within the thalamus (Victor et al., 2011).

By virtue of long-range axonal projections of excitatory cortical neurons and by assuming the spatially-homogeneous dynamics on the cortex, the average firing rate ϕ_*E*_ obeys the damped oscillator equation

(3) $$D\phi_{E}=S(V_{E}),$$

where the operator *D* is defined as

(4) $$D={\left(\frac{1}{\gamma }\frac{\partial }{\partial t}+1\right)}^{2},$$

and γ is the cortical damping rate. It is assumed that the spatial spread of activity is very fast in other populations and the activity variable can be approximated by a sigmoidal function as ϕ_*b*_ = *S*(*V_b_*), for *b* = *I, S, R*. Conversely to the original model (Robinson et al., 2001, 2002; Victor et al., 2011) we use a more realistic transfer function derived from properties of type I-neurons given by Hutt and Buhry (2014)

(5) $$S(V_{a})=Sig(V_{a},0)-Sig(V_{a},\rho ),$$

with

(6) $$\begin{array}{l} Sig(V_{a},\rho )=\frac{S_{a}^{max}}{2}\left(1+\text{erf}\left(\frac{V_{a}-\theta_{a}-\rho \sigma^{2}}{\sqrt{2}\sigma }\right)\right)\\ \;e^{-\rho (V_{a}-\theta_{a})+\rho^{2}\sigma^{2}/2}, \end{array}$$

with σ = 10 mV and ρ = 0.08 mV^−1^, where σ is related to the standard deviation of firing thresholds, the parameter ρ < ∞ reflects the properties of type I-neurons, *S*^*max*^ is the maximum population firing rate, and θ_*a*_ is the mean firing threshold of neurons in population *a*. In contrast to the standard transfer function given in Robinson et al. (2001), the transfer function in Equation (6) is not anti-symmetric to its inflection point anymore (Hutt, 2012) and exhibits a larger non-linear gain (slope) for large potentials *V_a_* > θ_*a*_ compared to small potentials *V_a_* < θ_*a*_. This asymmetry results from the firing properties of type-I neurons, see Hutt (2012); Hutt and Buhry (2014) for more details. For ρ → ∞, the sigmoid function becomes the conventional antisymmetric transfer function.

The external input to the system is considered as a non-specific input to thalamo-cortical relay neurons as

(7) $$\phi_{N}=\langle \phi_{N}\rangle +\sqrt{2\kappa }\xi (t),$$

where 〈ϕ_*N*_〉 indicates its mean value and ξ(*t*) is a zero average Gaussian white noise and κ is the noise intensity.

The power spectrum characterizes small fluctuations about the resting state of the system defined by *dV_a_*(*t*)/*dt* = 0. Following Robinson et al. (2001); Nunez and Srinivasan (2006), it is assumed that the activity of excitatory cortical neurons generates the EEG, and due to the specific choice of external input to thalamo-cortical relay neurons, the power spectrum of the EEG is related to the Greens function of linear deviations about the resting state by Hutt (2013)

(8) $$P_{E}(\omega )=2\kappa \sqrt{2\pi }{\left|{\tilde{G}}_{1,3}(\omega )\right|}^{2},$$

in which *P_E_*(ω) depends just on one matrix component of the matrix Greens function $\tilde{G}$(ω), see the Supplementary Material for its definition. We point out that the subsequent power spectrum analysis is based on Equation (8) and changing a system parameter, such as the factor *p*, changes the resting state, the corresponding non-linear gains and consequently the power spectrum. In addition, the power spectrum analysis is valid only if the resting state is stable and hence the fluctuations do not diverge. We have taken care of this additional condition and all given parameters guarantee the existence and stability of the resting state.

### 2.3. Effect of propofol on neural populations

In order to mimic anesthetic action, we consider the general anesthetic propofol which affects synaptic and extra-synaptic GABAergic receptors. We assume that the decay rate of inhibitory synapses is identical in all neural populations under study, and decreases with increasing propofol concentration in accordance with experimental findings (Kitamura et al., 2002; Hutt and Longtin, 2009). Mathematically, such a dependence on the anesthetic concentration can be taken into account by a concentration factor *p* ≥ 1 and β_*b*_ = β_0_/*p* while increasing *p* reflects an increase of the on-site concentration of propofol (Foster et al., 2008; Hindriks and van Putten, 2012; Hutt et al., 2013). Since propofol has been shown to retain the amplitude of inhibitory synaptic response functions (Kitamura et al., 2002), one can define *H_b_* = Γ(α, β_0_)/Γ(α, β_b_) for *b* = *I, R*, where

$$\Gamma (\alpha ,\beta )=\frac{\alpha \beta }{\alpha -\beta }\left[{\left(\alpha /\beta \right)}^{\frac{-\beta }{\alpha -\beta }}-{\left(\alpha /\beta \right)}^{\frac{-\alpha }{\alpha -\beta }}\right],$$

i.e., Γ(α, β_*b*_) = $\bar{h}$_*b*_(*t*_0_) is the peak amplitude of $\bar{h}$_*b*_(*t*) at time *t*_0_ = ln(α/β_*b*_)/(α − β_*b*_). Thereby the maximum height of *h*_*b*_(*t*) is *h*_*max*_ = Γ(α, β_0_) which is independent of the action of propofol (Hindriks and van Putten, 2012). Moreover, since it is assumed that propofol does not act on excitatory synaptic transmission, *H*_*b*_ = 1 and *h*_*b*_(*t*) = $\bar{h}$_*b*_(*t*) for *b* = *E, S*. The GABAergic ESR tonic inhibition can be represented in the model as a constant shift of the firing threshold in neural population models (Hutt and Buhry, 2014). For simplicity, we assume a linear relationship between the anesthetic concentration parameter *p* and the extra-synaptic threshold shift

(9) $$\theta_{a}=\theta_{0}+(p-1)k_{a}$$

with the unique firing threshold θ_0_ = 15 mV identical for all populations in the absence of propofol and the extra-synaptic anesthetic sensitivity *k_a_* > 0. Here, (*p* − 1)*k_a_* is the tonic inhibition induced by extra-synaptic action which depends linearly on the propofol concentration. Future experimental studies may motivate a more realistic relationship of threshold shift and the anesthetic concentration parameter. Summarizing, synaptic and extra-synaptic inhibition, and hence anesthetic action, is present in the cortical populations *E* and *I* and in the thalamic population of relay neurons *S*.

### 2.4. Power spectrum

The present study examines the effect of tonic inhibition in various populations *E, I, S* on the power spectrum of neural activity in cortical excitatory neurons, i.e., population *E*. We will focus on the power in the δ− and α− frequency ranges in the interval [0.5 Hz−4Hz] and [8 Hz−12 Hz], respectively.

The subsequent analysis reveals power peaks in these frequency ranges, whose magnitude changes with the level of tonic inhibition. These power peaks exhibit a maximum of power, expressed mathematically as a local maximum of the function *P_E_*(*f*) where *P_E_* is taken from Equation (8). The local maximum at frequency *f*_0_ is defined as *dP_E_*/*df* = 0, *d*^2^*P_E_*/*df*^2^ < 0 computed at *f*_0_. If there is a local maximum of power in the δ− frequency range, then δ−activity is present, whereas a missing local maximum in the δ−frequency range indicates missing δ−activity. Since the magnitude and frequency of power peaks change with the propofol concentration and extra-synaptic threshold, the concentration factor *p* and the extra-synaptic anesthetic sensitivity *k_a_* are the parameters of the power spectrum, i.e., *P_E_* = *P_E_*(*p, k_a_, f*).

To illustrate the usefulness of this parametrization, let us assume a factor *k*_*a*0_ for which no δ−power peak exists in the power spectrum *P_E_*(*p, k*_*a*0_, *f*), and *k*_*a*1_, *k*_*a*1_ > *k*_*a*0_ is the extra-synaptic anesthetic sensitivity leading to a spectral δ−power peak in *P_E_*(*p, k*_*a*1_, *f*) with *dP_E_*(*p, k*_*a*1_, *f_max_*)/*df_max_* = 0 where *f_max_* is a frequency in the δ−frequency range. Mathematically, then the continuity of all model functions and variables guarantee that there is a threshold for the emergence of δ−activity at a certain extra-synaptic anesthetic sensitivity *k_a, thr_* with *k*_*a*0_ ≤ *k_a, thr_* ≤ *k*_*a*1_. Consequently, if a threshold extra-synaptic anesthetic sensitivity for δ−activity exists, then the variation of model variables about this critical point guarantees the emergence of δ−activity. This mathematical reasoning allows us to investigate conditions under which δ−activity may emerge.

## 3. Results

**Figure 2:**
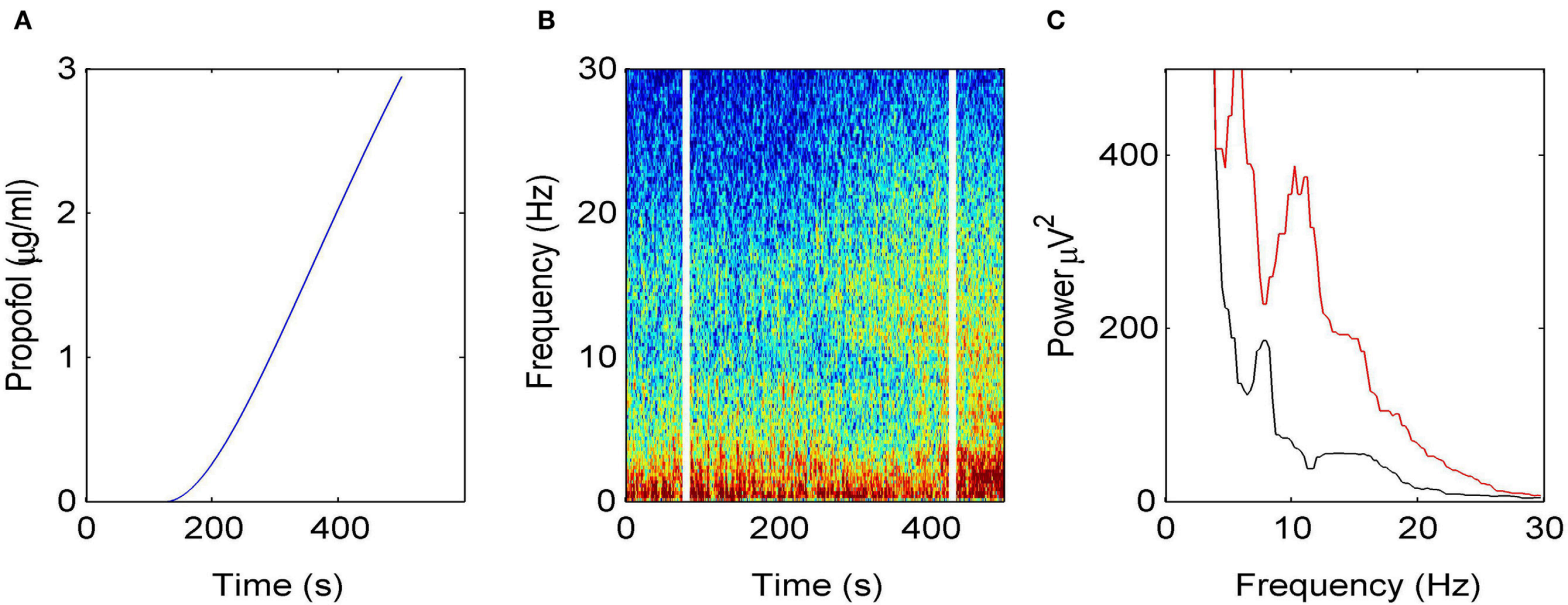
**Electroencephalographic data observed under anesthesia sedation in a single subject while increasing the propofol concentration. (A)** Blood plasma concentration of propofol with respect to administration time. **(B)** Spectrogram of frontal EEG power. The vertical lines denote time windows well before the administration (left line) and at about 5 min after the start of propofol infusion (right side); **(C)** Power spectra computed before the infusion of propofol (black) and 5 min after the start of infusion (red).

Figure 2 illustrates how the EEG power spectrum depends on the concentration of propofol for a single subject. After starting the infusion at *t* = 0 min, the estimated propofol effect-site concentration increases gradually with time (Figure 2A); resulting in increased power in the δ− and α−frequency ranges (Figure 2B). Over the period of the spectrogram the subject has become progressively more sedated; until a *t* = 5 min the subject no longer responds to verbal command but would still be responsive to nociceptive stimuli. Figure 2C shows the power spectra in the awake and sedation conditions. We observe a power enhancement primarily in the δ− and α−frequency ranges.

**Figure 3:**
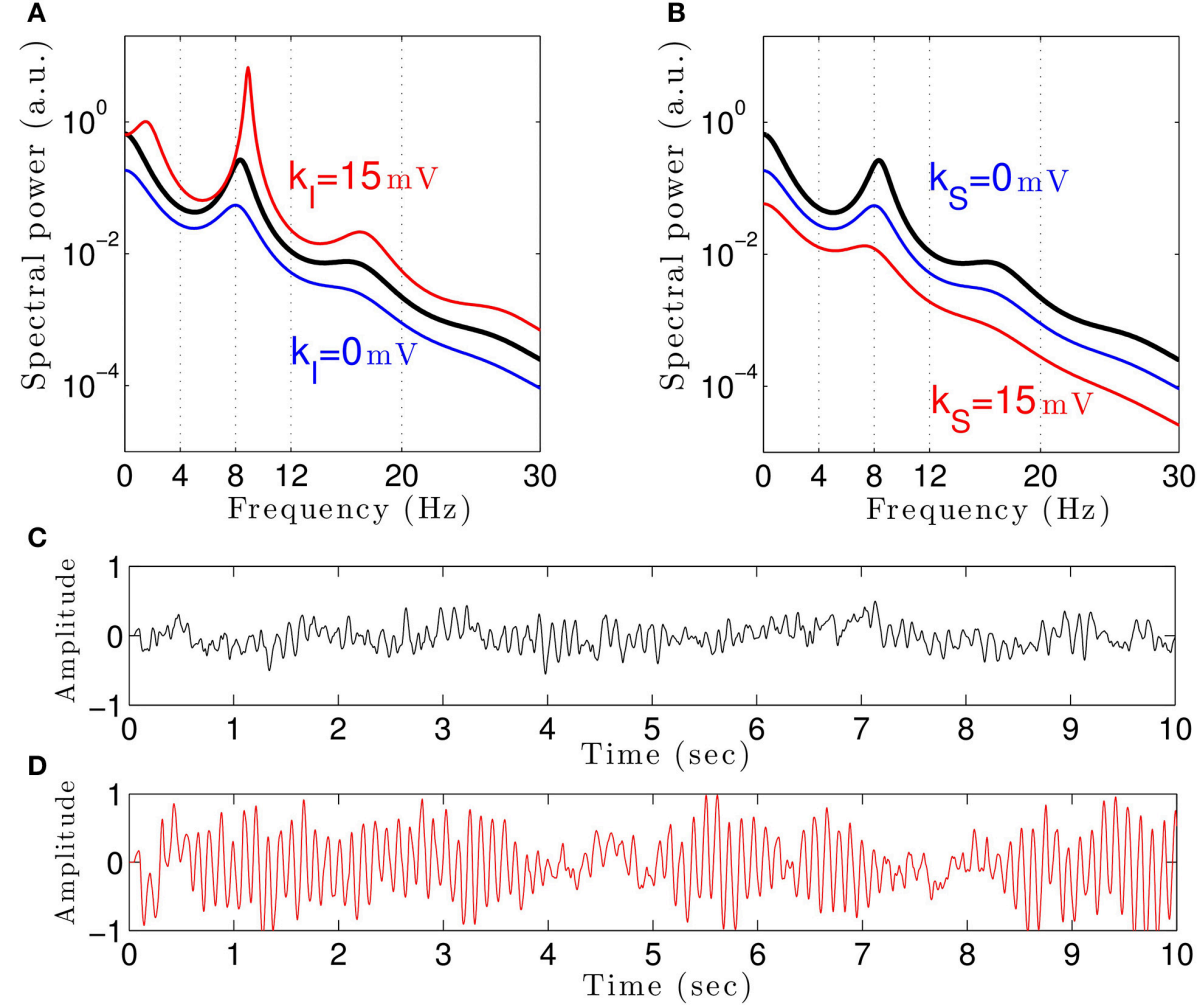
**The theoretical EEG power spectrum in the baseline and in the sedation condition with and without tonic inhibition in the cortical inhibitory neurons *I* in (A) and the thalamic relay neurons *S* in (B) and corresponding simulated EEG time-seris**. In **(A)** the administration of propofol without tonic inhibition (blue line) attenuates the power spectrum compared to the baseline condition (black line) while the tonic inhibition (red line) increases the global power and generates oscillatory activity in the δ-frequency range. In **(B)** increasing the anesthetic concentration yields a global power decrease in the sedation condition without tonic inhibition (blue line) and a further power decrease in the presence of tonic inhibition (red line). In **(A)** and **(B)**, the black lines indicate the EEG-spectral power in the baseline condition (*p* = 1), and the blue and red lines show the power spectrum in anesthesia condition (*p* = 1.125) in the absence (*k*_*a*_ = 0) and in the presence (*k_a_* = 15 mV) of tonic inhibition, respectively. **(C)** The simulated EEG time-series (ϕ_*E*_(*t*) defined in Equation (3)) in the absence of extra-synaptic effects, i.e., *k_E_* = *k_I_* = *k_S_* = 0 mV. **(D)** The EEG time-series in the presence of extra-synaptic action in cortical inhibitory neurons with *k_I_* = 15 mV, *k_E_* = *k_S_* = 0 mV. The tonic inhibition changes the EEGs from low-amplitude, high-frequency pattern to high-amplitude, low-frequency pattern. In addition, the strength of cortical self-inhibition is ν_*ii*_ = −1.8 mVs.

To understand how propofol might enhance δ− and α−power, we study the power spectrum of our theoretical model for different anesthetic concentration levels and examine the impact of adding tonic inhibition via extra-synaptic GABA_*A*_ receptors. Figure 3A shows the interaction between propofol and tonic inhibition in the cortical inhibitory neuronal population. If we set the tonic inhibition to zero (*k_I_* = 0 mV), we observe a decrease in spectral power as propofol concentrations increase (i.e., the power moves from the black line to the blue line in the figure). If we set the tonic inhibition to (*p* − 1) · 15 mV we see the opposite effect—there is an increase of δ− and α−power (black line to red line), with increasing propofol concentration.

Previous studies have indicated that extra-synaptic inhibition in thalamic relay neurons may control the level of inhibition in the brain (Brickley and Mody, 2012). However, Figure 3B reveals that adding a nonzero tonic inhibition in the thalamic relay neurons causes a decrease in the spectral power, similar to the previous case of absent tonic inhibition in the inhibitory cortical neurons.

It is well-known that GABAergic anesthetics change the EEG from high frequency-low amplitude signals to low frequency-high amplitude signals (Gugino et al., 2001; Feshchenko et al., 2004). Figures 3C,D show simulated time series in the absence and presence of tonic inhibition in cortical inhibitory cells reproducing this experimental finding.

**Figure 4:**
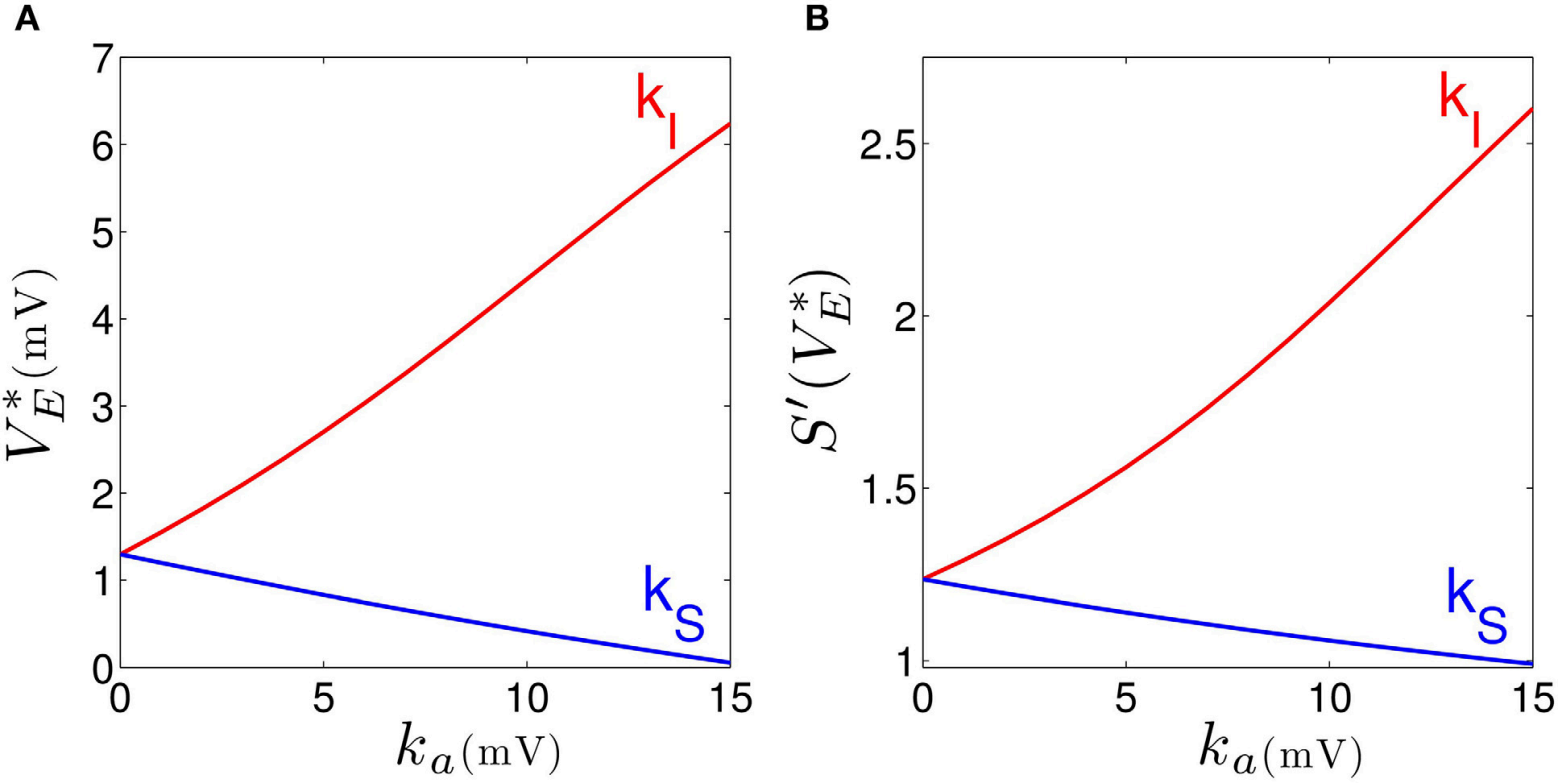
Increasing the tonic inhibition (factor *k_a_* for *a* = *I* and *S*) affects the resting state of excitatory cortical neurons V^*^_E_ (A) and the corresponding non-linear cortical gain function (B). Here the anesthetic concentration factor is identical in the populations *a* = *E, I* and *S* to *p* = 1.125. In addition, the strength of cortical self-inhibition is ν_*ii*_ = −1.8 mVs.

Our results elucidates that tonic inhibition in cortical interneurons and thalamic relay neurons affect the cortical power spectrum differently. This finding is similar to results of a previous computational neural population study of a cortico-thalamic feedback single-neuron model (Talavera et al., 2009). Figure 4 shows how the resting membrane potential (Figure 4A) and the non-linear gain (Figure 4B) in the cortical excitatory population change with differing extra-synaptic anesthetic sensitivity in cortical inhibitory neurons (*k_I_*) and in the thalamic relay neurons (*k_S_*). We observe that both the resting potential and the non-linear gain of cortical excitatory neurons increase when the cortical inhibitory extra-synaptic anesthetic sensitivity *k_I_* increases, whereas resting potential and non-linear gain of cortical excitatory neurons decrease when the extra-synaptic anesthetic sensitivity in thalamic relay neuron *k_S_* increases. Since the non-linear gain is proportional to the systems responsiveness to external stimuli, the power enhancement in population *I* may be explained by the augmented responsiveness of the cortical excitatory neurons. This responsiveness depends on the sub-circuit in which the neurons are involved. Since relay neurons are part of the thalamo-cortical feedback loop, while cortical inhibitory neurons contribute to the cortical loop, the cell types respond differently to the thalamic input. Essentially assuming tonic inhibition in the population of cortical excitatory neurons *E*, the study reveals a similar propofol concentration dependence of the power spectrum, the resting state potential and the non-linear gain as for the thalamic tonic inhibition *S*. This shows the unique tonic inhibition effect in the cortical inhibitory neurons.

**Figure 5:**
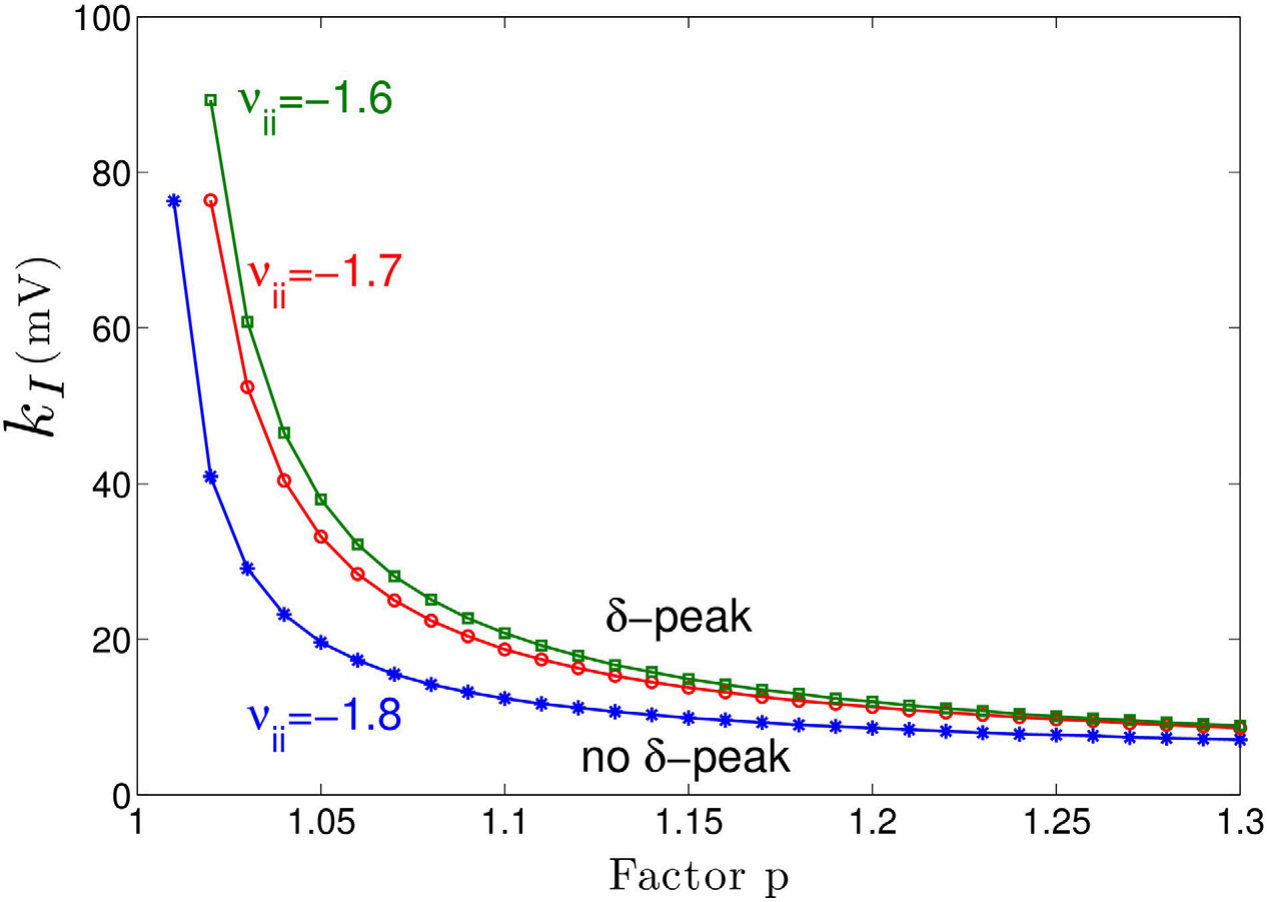
**Parameter space for δ-power peak**. The lines give the smallest (threshold) value of the extra-synaptic sensitivity *k_I, thr_* that induces δ− oscillations in the sedation condition with respect to the concentration factor *p* for different values of self-inhibitory connections ν_*ii*_. The weaker the cortical self-inhibition (the smaller |ν_*ii*_|), the higher the necessary level of propofol concentration (larger *p*) and the tonic inhibition [larger (*p* − 1) · *k_I_*] to induce δ−activity.

Figure 3A shows the power spectrum for single values of the extra-synaptic sensitivity *k_I_*, for single values of the concentration factor *p* and fixed strength of cortical self-inhibition ν_*ii*_, while Figure 4 gives more details on the role of extra-synaptic sensitivity for fixed values of the concentration factor *p* and fixed cortical self-inhibition. To understand better the interplay between tonic inhibition, synaptic inhibition and the strength of cortical self-inhibition, Figure 5 shows the parameter pairs of synaptic inhibition *p* and the threshold of extra-synaptic sensitivity *k*_*I, thr*_ at different self-inhibition levels, for which a peak in the δ−frequency range emerges. Recall that the *k_I, thr_* is the critical (smallest) value of extra-synaptic sensitivity in cortical inhibitory neurons *k_I_*, that lead to *dP_E_*/*df* = 0, *d*^2^*P_E_*/*df*^2^ < 0 computed at *f_max_* ∈ δ−range, cf. the subsection on the power spectrum in Section 2. Parameter values beyond the respective curves lead to δ−activity power peaks. We observe that δ−activity always emerges for sufficiently strong tonic inhibition (large extra-synaptic sensitivity *k_I_*) and sufficiently strong self-inhibition ν_*ii*_, while the weaker the self-inhibition is the larger is the necessary extra-synaptic sensitivity or the synaptic inhibition to generate δ−activity. Even for vanishing cortical self-inhibition (ν_*ii*_ = 0), mathematical analysis (not shown) reveals that there is still a δ−peak in the power spectrum for large enough synaptic or tonic inhibition (for *p* or *k_I_* large enough).

Moreover, Figure 5 reveals a minimum tonic inhibition level (minimum value of *k_I_*) beneath which no δ−power peak emerges, irrespective of the level of synaptic inhibition (*p*). This result indicates a major role of tonic inhibition in the generation of δ−activity, since it may support δ−activity even if the synaptic inhibition level is not sufficient to support it.

## 4. Discussion

In the sedation phase, for modest concentrations of propofol, the EEG power spectrum exhibits an increase in the δ− and α−frequency ranges (Figure 2) as found experimentally in the induction phase of propofol anesthesia (San-Juan et al., 2010). One possible explanation for these phenomena is by postulating stronger GABAergic potentiation within cortical inhibitory neurons than within cortical pyramidal neurons (Hindriks and van Putten, 2012). We hypothesize, that cortical GABAergic self-inhibition plays a decisive role. Figure 3 reveals that the power surge in these frequency ranges might also result from extra-synaptic tonic inhibition active in cortical inhibitory neurons. Tonic inhibition increases the firing threshold and hence diminishes the output of inhibitory neurons to excitatory cortical neurons, which then allows increased excitation in the excitatory population and a power surge in the EEG. Conversely tonic inhibition in the thalamic relay cells does not induce power surge in EEG since augmented inhibition in the thalamic relay cells yields diminished excitation in cortical excitatory neurons, leading to a decrease in EEG power. This interpretation is corroborated by Figure 4 which demonstrates augmented and diminished non-linear gain in cortical excitatory neurons assuming tonic inhibition in inhibitory and thalamic relay population, respectively. This reflects enhanced and weakened response to the noisy thalamic external input, see previous theoretical studies (Hindriks and van Putten, 2012; Hutt, 2013) for a similar line of argument.

Figure 3 clearly reveals the emergence of δ−activity caused by extra-synaptic tonic inhibition which is affirmed by the existence of a minimum level of extra-synaptic inhibition shown in Figure 5. Conversely, α−activity appears to be much less sensitive to tonic inhibition since it is present for all tonic inhibition levels. One interpretation may be the generation of α−activity by the cortico-thalamic feedback as hypothesized theoretically (Robinson et al., 2004) while δ−activity results from the cortical interaction of excitatory and inhibitory neurons. The exact origins of propofol-induced α− and δ−activity are not known for certain. We find that the α−oscillations arise from thalamocortical resonances. These oscillations are commonly synchronous across widespread cortical regions and are not easily generated in isolated cortical tissue (Contreras et al., 1996; Destexhe et al., 1999). This affirms the original model of Lopes da Silva et al., (1974). However, our model results are equivalent to results of other models describing α−activity by purely cortical interactions. We are not aware of a methodology ruling out one or the other model and this is not the aim of the present work. Our work just reveals the additional possibility that the thalamus serves as a possible (indirect) source of α−activity. Similarly, the origin of δ−activity is not clear, but slow activity does increase at higher concentrations of propofol—which may be associated with decreasing α−waves as observed during desflurane general anesthesia (Mulholland et al., 2014). This is in keeping with δ−waves becoming more pronounced as the cortico-thalamic systems becomes increasingly hyperpolarized. However, there is a lot of variability between patients as regards the relative power of α− and δ−activity during general anesthesia; which would suggest that the true explanation is more complex, and requires recognition of other factors such as the one presented in this paper—the influence of the propofol on extra-synaptic inhibition.

Although anesthetic action on synaptic and extra-synaptic GABAergic receptors is different, both actions diminish neural activity and hence increase inhibition. Figure 5 elucidates that strong enough extra-synaptic or synaptic inhibition induce δ−activity. Hence, one may argue that the level of inhibition plays an important role while its origin, i.e., synaptic or extra-synaptic, plays a secondary role. This interpretation corroborates the idea of the balance of excitation and inhibition as the major mechanism in general anesthesia. This interpretation is in good accordance to previous experimental findings on the important role of the balance of excitation and inhibition in brain network under anesthesia (Okun and Lampl, 2008; Taub et al., 2013). Such global concepts as excitation-inhibition balance are attractive to describe complex processes in general anesthesia. For instance, anesthetics alter arousal in several pathways, such as the cholinergic pathway (Brown et al., 2010) and the orexinergic pathway which has been identified to activate a complex functional network controlling, inter alia, the emergence from unconsciousness (Kelz et al., 2008).

Our theoretical study considers the anesthetic propofol and its corresponding action at synaptic and extra-synaptic GABAergic receptors only, whereas it is known that propofol induces inhibition at various other receptors as well (Alkire et al., 2008; Nguyen et al., 2009) including minor effects on NMDA-receptors and voltage-gated potassium channels (Alkire et al., 2008). Propofol also potentiates glycine receptors which are are found all over the central nervous system and have a major role in regulating inhibition, e.g., in the brain stem (Lynch, 2004).

Similar to extra-synaptic inhibition resulting from ambient GABA concentrations, the presence of ambient concentrations of glycine close to NMDA-receptors entails tonic depolarization. This tonic excitation diminishes the firing threshold of neurons and hence may counteract inhibition. The present work considers tonic inhibition only and neglects tonic excitation effect. Although it would be important to study tonic excitation effects, this additional study would exceed the major aim of the manuscript, namely demonstrating the fundamental effect of tonic anesthetic action.

In addition, by virtue of the focus on extra-synaptic action, the model proposed neglects known anesthetic effects on different receptors and ion channels, although they have been shown experimentally, e.g., Grasshoff et al. (2006); Alkire et al. (2008) and references therein, and theoretically (Bojak et al., 2013) to affect EEG activity. Specifically, the latter work of Bojak et al. (2013) considers anesthetic effects on hyperpolarization-activated cyclic nucleotide-gated potassium channel 1 (HCN1) subunits which, effectively, increase the mean firing threshold in neural populations and strongly resembles the tonic inhibition induced by extra-synaptic GABA-receptors.

The model network topology includes a single module of a closed thalamo-cortical feedback loop (Granger and Hearn, 2007) comprising two thalamic nuclei and cortical excitatory and inhibitory neurons. This model represents a first approximation of brain networks since it neglects brain stem activity including the reticular activating system (RAS) (Magoun, 1952) which has significant modulating effects on attention, arousal and consciousness. Future work will include structures of the brain stem, propofol action on glycine receptors, and will take into account the RAS—since its neural structures involved exhibit strong extra-synaptic inhibition (Fiset et al., 1999; Franks, 2008; Vanini and Baghdoyan, 2013). The model also neglects the cholinergic pathway originating from the basal forebrain (Laalou et al., 2008) which is known to co-regulate the level of consciousness (Brown et al., 2010).

Essentially, our theoretical model assumes population coding implying rate-coding response activity of neuron populations subjected to external thalamic noise. The model does not consider specific single neuron dynamics found experimentally under anesthetic conditions. For instance, it has been hypothesized that, at certain levels of anesthetic concentration, thalamic neurons switch their activity from tonic firing to bursting and induce loss of consciousness (Alkire et al., 2000).

In spite of these limitations, our model reproduces qualitatively the action of propofol on EEG and reveals the possible impact of extra-synaptic GABAergic receptors on the EEG power. To our knowledge, the present work is the first to link extra-synaptic GABAergic action and experimental EEG. Future work will refine the model involving additional receptor action, e.g., tonic excitation caused by ambient glycine concentrations, and sub-cortical brain structures.

## Author contributions

Meysam Hashemi has chosen the model and performed the model analysis, Axel Hutt has conceived the study and Jamie Sleigh has acquired and analyzed the experimental data. The authors have written the manuscript to equal parts and have approved the final version.

## Funding

The research resulting to the presented work has received funding from the European Research Council under the European Unions Seventh Framework Programme (FP7/2007-2013) / ERC grant agreement no. 257253.

### Conflict of interest statement

The authors declare that the research was conducted in the absence of any commercial or financial relationships that could be construed as a potential conflict of interest.
