# Supplementary material for: Anesthetic action on extra-synaptic receptors: effects in neural population models of EEG activity
Source: Front Syst Neurosci. 2014 Dec 10;8:232. doi: 10.3389/fnsys.2014.00232 (PMC4261904; doi:10.3389/fnsys.2014.00232)
Supplement: Supplementary file 1 [file DataSheet1.PDF]

APPENDIX A. POWER SPECTRUM

The resting state (stationary state) of Eqs. (1) defined by $dV_a(t)/dt = 0$ obeys

$$\begin{aligned}
V_E^* &= \nu_{ee}S(V_E^*) + H_b\nu_{ei}S(V_I^*) + \nu_{es}S(V_S^*),\\
V_I^* &= \nu_{ie}S(V_E^*) + H_b\nu_{ii}S(V_I^*) + \nu_{is}S(V_S^*),\\
V_S^* &= \nu_{se}S(V_E^*) + H_b\nu_{sr}S(V_R^*) + \langle\phi_N\rangle,\\
V_R^* &= \nu_{re}S(V_E^*) + \nu_{rs}S(V_S^*),
\end{aligned}$$

where V_a^* denotes the resting state value of V_a for $a = E, I, S, R$. Moreover Eq. (3) gives $\phi_E^* = S(V_E^*)$. Then we linearize Eqs. (1) about the obtained resting state and write them in a general matrix form of a linear DDE as

$$\hat{\boldsymbol{L}}\left(\frac{\partial}{\partial t}\right)\boldsymbol{X}(t) = \boldsymbol{A}\boldsymbol{X}(t) + \boldsymbol{B}\boldsymbol{X}(t-\tau) + \boldsymbol{\xi}(t),$$

where

$$\boldsymbol{X}(t) = \begin{pmatrix} \phi_E(t) - \phi_E^* \\ V_I(t) - V_I^* \\ V_S(t) - V_S^* \\ V_R(t) - V_R^* \end{pmatrix}, \quad \hat{\boldsymbol{L}} = \begin{pmatrix} \tilde{L}\frac{\tilde{D}}{K_{11}} & 0 & 0 & 0 \\ 0 & \tilde{L} & 0 & 0 \\ 0 & 0 & \tilde{L} & 0 \\ 0 & 0 & 0 & \tilde{L} \end{pmatrix},$$

$$\boldsymbol{A} = \begin{pmatrix} K_1 & K_2 & 0 & 0 \\ K_4 & K_5 & 0 & 0 \\ 0 & 0 & 0 & K_8 \\ 0 & 0 & K_{10} & 0 \end{pmatrix}, \quad \boldsymbol{B} = \begin{pmatrix} 0 & 0 & K_3 & 0 \\ 0 & 0 & K_6 & 0 \\ K_7 & 0 & 0 & 0 \\ K_9 & 0 & 0 & 0 \end{pmatrix}, \quad \boldsymbol{\xi}(t) = \begin{pmatrix} 0 \\ 0 \\ \sqrt{2\kappa}\xi(t) \\ 0 \end{pmatrix},$$

with $\tilde{L} = (1 + i\omega/\alpha)(1 + i\omega/\beta_0)$, $\tilde{D} = (1 + i\omega/\gamma)^2$, and

$$\begin{aligned}
K_1 = \nu_{ee}\,, K_2 = H_b\nu_{ei}\frac{\partial S_I[V]}{\partial V}\,|_{V=V_I^*}\ , \ K_3 = \nu_{es}\frac{\partial S_S[V]}{\partial V}\,|_{V=V_S^*},\\
K_4 = \nu_{ie}\ , \ K_5 = H_b\nu_{ii}\frac{\partial S_I[V]}{\partial V}\,|_{V=V_I^*}\ , \ K_6 = \nu_{is}\frac{\partial S_S[V]}{\partial V}\,|_{V=V_S^*},\\
K_7 = \nu_{se}\ , \ K_8 = H_b\nu_{sr}\frac{\partial S_R[V]}{\partial V}\,|_{V=V_R^*}\ , \ K_9 = \nu_{re},\\
K_{10} = \nu_{rs}\frac{\partial S_S[V]}{\partial V}\,|_{V=V_S^*}\ , \ K_{11} = \frac{\partial S[V]}{\partial V}\,|_{V=V_E^*}\,.
\end{aligned}$$

The power spectral density matrix $\boldsymbol{P}(\omega)$ of $\boldsymbol{X}(t)$ is the Fourier transform of the auto-correlation function matrix $\langle \boldsymbol{X}(t)^t\boldsymbol{X}(t-T)\rangle$ (Wiener-Khinchine Theorem) leading to

$$\boldsymbol{P}(\omega) = 2\kappa\sqrt{2\pi}\tilde{\boldsymbol{G}}(\omega)\tilde{\boldsymbol{G}}^t(-\omega),$$

where $\tilde{\boldsymbol{G}}(\omega)$ is the Fourier transform of the matrix Greens function and the high index t denotes the transposed matrix (25).

At the end, the model assumes that excitatory activity generates the EEG and by virtue of the specific choice of external input to relay neurons, the power spectrum of the EEG depends just on one matrix component of the Greens function by

$$P_E(\omega) = 2\kappa\sqrt{2\pi}\tilde{G}_{1,3}(\omega)\tilde{G}_{1,3}(-\omega) = 2\kappa\sqrt{2\pi}\left|\tilde{G}_{1,3}(\omega)\right|^2,$$

cf. Eq. (8), where

$$\tilde{\boldsymbol{G}}(\omega) = \frac{1}{\sqrt{2\pi}}[\hat{\boldsymbol{L}} - \boldsymbol{A} - \boldsymbol{B}e^{-i\omega\tau}]^{-1} = \frac{1}{\sqrt{2\pi}}\begin{bmatrix} \tilde{L}\frac{\tilde{D}}{K_{11}} - K_1 & -K_2 & -K_3e^{-i\omega\tau} & 0 \\ -K_4 & \tilde{L} - K_5 & -K_6 & 0 \\ -K_7e^{-i\omega\tau} & 0 & \tilde{L} & -K_8 \\ -K_9e^{-i\omega\tau} & 0 & -K_{10} & \tilde{L} \end{bmatrix}^{-1}.$$
