# Supplementary material for: Anesthetic action on extra-synaptic receptors: effects in neural population models of EEG activity
Source: Front Syst Neurosci. 2014 Dec 10;8:232. doi: 10.3389/fnsys.2014.00232 (PMC4261904; doi:10.3389/fnsys.2014.00232)
Supplement: Supplementary file 2 [file DataSheet2.PDF]

APPENDIX B. PARAMETER VALUES

The subsequent table gives the nominal values of model parameters.

Parameter	Symbol	Nominal value
Maximum firing-rate of all populations	S^{max}	250 Hz
Mean firing threshold of all populations	θ	15 mV
Firing rate variance of all populations	σ	10 mV
Type-I population effect constant of all populations	ρ	0.08 mV^{-1}
Synaptic rise rate	α	200 s^{-1}
Synaptic decay rate	β_0	50 s^{-1}
Synaptic strength from E to E neurons	ν_{ee}	1.2 mVs
Synaptic strength from E to I neurons	ν_{ie}	1.2 mVs
Synaptic strength from E to S neurons	ν_{se}	1.2 mVs
Synaptic strength from E to R neurons	ν_{re}	0.4 mVs
Synaptic strength from I to I neurons	ν_{ii}	-1.8 mVs
Synaptic strength from I to E neurons	ν_{ei}	-1.8 mVs
Synaptic strength from S to E neurons	ν_{es}	1.2 mVs
Synaptic strength from S to I neurons	ν_{is}	1.2 mVs
Synaptic strength from S to R neurons	v_{rs}	0.2 mVs
Synaptic strength from R to S neurons	ν_{sr}	-0.8 mVs
Mean value of external input	$\langle\phi_N\rangle$	1 mV
Intensity of external noise	κ	0.1 mV
Cortical damping rate	γ	150 s^{-1}
Transmission delay between cortex and thalamus	τ	40 ms
